# PYRAZINAMIDE-INDUCED PHOTOTOXICITY: A CASE REPORT AND REVIEW OF LITERATURE

**DOI:** 10.4103/0019-5154.60368

**Published:** 2010

**Authors:** Subodh K Katiyar, Shailesh Bihari, Shivesh Prakash

**Affiliations:** *From the Department of Tuberculosis and Respiratory Diseases, GSVM Medical College, Kanpur, India.*

**Keywords:** *Antitubercular drugs*, *pyrazinamide*, *phototoxicity*

## Abstract

A 40-year-old male presented with a fresh case of pulmonary tuberculosis and itchy oozing rashes distributed characteristically over the sun exposed areas of the skin. These rashes had developed since six days following 10 days of start of antitubercular drugs (streptomycin, isoniazid, rifampicin, pyrazinamide and ethambutol at standard dosages). A possibility of drug-induced reaction was entertained and all the antitubercular drugs were discontinued; subsequently they were reintroduced in a sequential manner starting with small dosages, gradually increasing them to their normal dose. The rashes reappeared after introduction of pyrazinamide. We tried to desensitize this very important antitubercular drug but were not successful as the rashes reappeared. The patient was labeled as having pyrazinamide-induced phototoxicity and was started on a regimen containing streptomycin, isoniazid, rifampicin, ethambutol. Five months following treatment, the patient is now sputum negative for AFB. Pyrazinamide forms the integral part of most of the short course regimens, included in all the three categories of DOTS and with increasing coverage of DOTS therapy these rare cases may well be frequently encountered.

## Introduction

Tuberculosis still remains a worldwide public health problem despite the fact that its causative organism was discovered more than 100 years ago. According to conservative estimates, there are 15-20 million cases of infectious tuberculosis worldwide. This infectious pool is maintained by the occurrence of eight to nine million new cases and three million deaths each year. The magnitude of the problem has been such that the World Health Organization (WHO) had to declare it a global emergency in 1993. Pyrazinamide (PZA) forms an integral part of most of the short course regimens, included in all the three categories of DOTS.[1] Toxic effects that are common to PZA are hepatitis and arthralgia, hypersensitivity reactions are very rare with this drug.[2] With the increasing use of anti-tubercular drugs (ATD), one is likely to encounter hypersensitivity reactions with this drug in clinical practice more often. We report a case of PZA-induced hypersensitivity in this case report.

## Case Report

**Figure 1:**
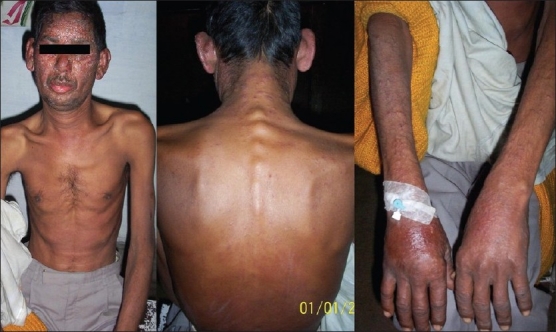
Phototoxicity rash seen over exposed areas

**Figure 2:**
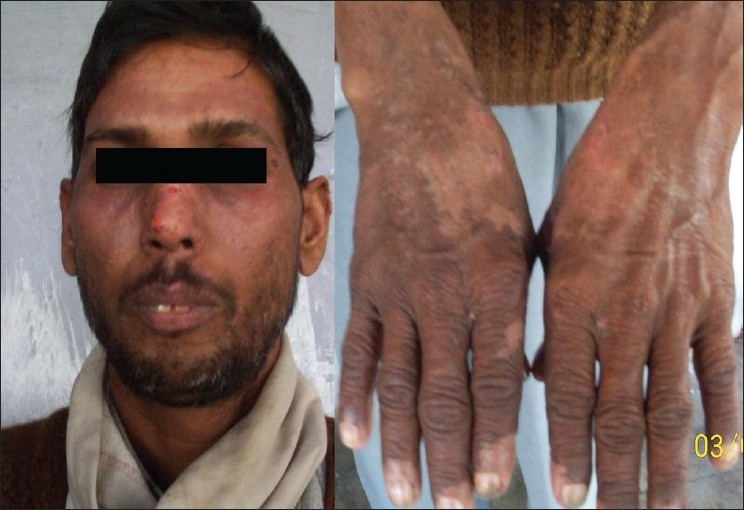
Patient improved after withdrawal of pyrazinamide

A 40-year-old farmer, father of two kids, chronic smoker and a resident of an urban area presented to us with chief complaints of itchy oozing rash since six days following 10 days of start of antitubercular chemotherapy comprising of streptomycin (S), isoniazid (INH), rifampicin (RIF), pyrazinamide (PZA) and ethambutol (E). His other complaints where productive cough, low grade fever, exertional breathlessness and left sided chest pain since three months. As evident from a positive sputum smear for acid-fast bacilli (AFB) with radiological evidence, he was a known case of pulmonary tuberculosis. On examination, there were erythematous maculo-papular rashes with oozing serous discharge along with generalized edema, distributed characteristically over the sun exposed areas of the skin [Figure 1]. A possibility of drug-induced hypersensitivity reaction was entertained and all the ATD were discontinued and he was managed using antibiotics, anti-histaminics, steroids, hydration and avoidance of sunlight. After six days of the above intervention, the rash subsided and anti-tubercular drugs were re-introduced one by one under close observation and steroids were tapered. E was introduced first at a low dose of 200 mg. Over the next four days the dose was escalated to 800 mg with no adverse event and hence continued. Then PZA was introduced at low dose of 250 mg which was escalated to 500 mg the next day. However, by the evening, he experienced recurrence of his previous rash which became prominent, erythematous and started itching, whereupon PZA was stopped and E continued. After a few days, the rash again subsided, when RIF was introduced at a low dose of 150 mg, which was escalated to 450 mg over next 4 days without any adverse event. This was followed by addition of INH at low dose of 100 mg escalated to 300 mg over four days without any adverse event. Hence, after about three weeks, he was finally stable on RIF 450 mg, INH 300 mg and E 800 mg [Figure 2]. After another five days, introduction of PZA (250 mg) was again attempted, which resulted in recurrence of skin eruptions on the same day. Patient was labelled as having PZA-induced phototoxicity and was started on a regimen containing S, INH, RIF and E. Five months following treatment, the patient is now sputum negative for AFB.

## Discussion

PZA proved to be the offending drug in the above case on the basis of the disappearance of rashes on withdrawal of the drug and subsequent reappearance on the re-administration of a test dose on two occasions.

There are very few reports of PZA-induced rashes in literature.[3–5] Allergic skin reactions to PZA are usually mild and liable to occur in approximately three per cent of cases.[6] Simultaneous hypersensitivity to S, PAS and INH can occurs in five per cent of all adverse reactions.[2] Hypersensitivity reactions are more common to other ATDs such as S, PAS and thiacetazone.[7] But, according to a report, incidence of PZA-induced hepatotoxicity and rash during treatment for active TB was substantially higher than with the other first-line ATDs, and higher than previously recognized.[8] The mechanisms behind PZA-induced phototoxicity was studied by Vargas *et al*.[9] They found that the phototoxic ATD PZA is photolabile under irradiation with UV-A light as well as with a N2 laser (at 337 nm) in aerobic conditions. According to a report, fever may be the sole manifestation,[2] initially, which is usually attributed either to the disease or to an incidental infection, but according to Toman,[10] it is always advisable that any new fever or a sudden increase of fever occurring within four weeks of starting chemotherapy should be suspected to be due to an allergic reaction, unless there is some other obvious reason but in our case it was not present.

Like in our case, most allergic reactions occur within the first four weeks of therapy and mainly present with a fever and/or skin rash. According to a few reports with PZA, desensitization is usually successful,[5] but if desensitization is carried out, it must be done under cover of at least two other ATDs to prevent emergence of drug resistance.[2]

If initial hypersensitivity is severe, desensitization should be best avoided, and if done, the process should be very slow with smaller doses.[11–13] We tried to reintroduce PZA but were not successful. Further attempts were not made as the initial reaction was severe and reappearance of rash was immediate. If the patient is restarted on an adequate tuberculosis treatment regimen, defined as three or more drugs of which at least two are bactericidal (RIF and INH), re-challenging with the implicated drug is not advisable.[13]

INH is the most potent ATD and RIF has a better sterilizing action than PZA. E prevents development of drug resistance, a property also shared by bactericidal drug S. PZA is a very limited agent in terms of its *in vitro* activity, but by virtue of its sterilizing action on dormant bacilli which are responsible for relapse, it facilitates reduction in duration of treatment from 9 to 6 months.[14] Hence good cure rates are possible with regimens without PZA, provided a minimum of nine months of treatment is ensured to prevent relapses.
